# *Lactobacillus reuteri* Inhibition of Enteropathogenic *Escherichia coli* Adherence to Human Intestinal Epithelium

**DOI:** 10.3389/fmicb.2016.00244

**Published:** 2016-03-01

**Authors:** Alistair D. S. Walsham, Donald A. MacKenzie, Vivienne Cook, Simon Wemyss-Holden, Claire L. Hews, Nathalie Juge, Stephanie Schüller

**Affiliations:** ^1^Norwich Medical School, University of East AngliaNorwich, UK; ^2^Gut Health and Food Safety Programme, Institute of Food ResearchNorwich, UK; ^3^Department of Gastroenterology, Norfolk and Norwich University HospitalNorwich, UK; ^4^Department of Surgery, Norfolk and Norwich University HospitalNorwich, UK; ^5^School of Biological Sciences, University of East AngliaNorwich, UK

**Keywords:** *L. reuteri*, EPEC, diarrhea, probiotic, human intestinal epithelium, adherence, mucus

## Abstract

Enteropathogenic *Escherichia coli* (EPEC) is a major cause of diarrheal infant death in developing countries, and probiotic bacteria have been shown to provide health benefits in gastrointestinal infections. In this study, we have investigated the influence of the gut symbiont *Lactobacillus reuteri* on EPEC adherence to the human intestinal epithelium. Different host cell model systems including non-mucus-producing HT-29 and mucus-producing LS174T intestinal epithelial cell lines as well as human small intestinal biopsies were used. Adherence of *L. reuteri* to HT-29 cells was strain-specific, and the mucus-binding proteins CmbA and MUB increased binding to both HT-29 and LS174T cells. *L. reuteri* ATCC PTA 6475 and ATCC 53608 significantly inhibited EPEC binding to HT-29 but not LS174T cells. While pre-incubation of LS174T cells with ATCC PTA 6475 did not affect EPEC attaching/effacing (A/E) lesion formation, it increased the size of EPEC microcolonies. ATCC PTA 6475 and ATCC 53608 binding to the mucus layer resulted in decreased EPEC adherence to small intestinal biopsy epithelium. Our findings show that *L. reuteri* reduction of EPEC adhesion is strain-specific and has the potential to target either the epithelium or the mucus layer, providing further rationale for the selection of probiotic strains.

## Introduction

Enteropathogenic *Escherichia coli* (EPEC) was first reported by Bray (1945) as a cause of infant summer diarrhea in the UK, and has since remained an important pathogen, particularly in the developing world (Nataro and Kaper, 1998). According to a recent systematic review, EPEC is the second most common cause (after rotavirus) of diarrheal death in children <5 years in the world (Lanata et al., 2013). One of the major virulence traits of EPEC is its ability to adhere to small intestinal epithelium by forming attaching/effacing (A/E) lesions. These are characterized by intimate bacterial attachment to the host cell membrane, effacement of underlying microvilli and polymerization of filamentous actin underneath adherent bacteria (Moon et al., 1983; Knutton et al., 1989). A/E lesion formation causes a loss of absorptive surface and, together with other effects mediated by type 3 secreted EPEC effector proteins, leads to the development of diarrhea (Viswanathan et al., 2009; Wong et al., 2011).

Clinical studies have shown that probiotic bacteria, such as lactobacilli can protect against intestinal infection (Sazawal et al., 2006). In particular, *Lactobacillus reuteri*, a natural inhabitant of the gastrointestinal tract of many mammals and birds (Casas and Dobrogosz, 2000), is effective as a therapeutic agent in acute rotavirus diarrhea in children (Shornikova et al., 1997a,b) and has recently been shown to protect against EPEC infection in infants (Savino et al., 2015). Several mechanisms of how probiotic bacteria protect against pathogens have been suggested and confirmed by *in vitro* studies. These include pathogen inhibition through microbe–microbe interactions (e.g., competition for nutrients and binding sites, production of antimicrobials), enhancement of epithelial barrier function (e.g., induction of mucins and preservation of tight junctions), and modulation of immune responses (e.g., regulation of cytokine expression, phagocyte, and T cell function; Lebeer et al., 2008). Competition for binding sites, also referred to as competitive exclusion, has been the focus of many *in vitro* studies (Bernet et al., 1994; Forestier et al., 2001; Sherman et al., 2005) and forms part of the rationale why adherence to intestinal mucosa is considered a desirable trait for probiotic bacteria (Havenaar et al., 1992; Tuomola et al., 2001). However, most of these studies have been performed on human enterocyte-derived cell lines (e.g., Caco-2, HT-29, T84) which do not produce a secreted mucus layer (van Klinken et al., 1996; Navabi et al., 2013). While this approach might be suitable to study the effects of probiotics on inflammatory bowel disease or other conditions with a compromised mucus layer (Sheng et al., 2012), it remains unknown whether findings on non-mucus producing epithelial cell lines can be translated to a normal intestinal mucosa where the epithelium is protected by a thick mucus layer (Juge, 2012).

In this study, we have investigated the effect of *L. reuteri* on EPEC adherence to mucus- and non-mucus-secreting human intestinal epithelial cell lines (LS174T and HT-29 cells, respectively) and to human small intestinal explants in an *in vitro* organ culture model.

## Materials and Methods

### Cell Culture

Human colon carcinoma HT-29 (ATCC HTB-38) and LS174T cells (ATCC CL-188) were cultured in DMEM medium (Sigma) supplemented with 10% fetal bovine serum (Sigma) and used between passage 5 to 20 and passage 7 to 27, respectively. Cells were grown at 37°C in a 5% CO_2_ atmosphere.

### Bacterial Strains and Culture Conditions

Bacterial strains used in this study are listed in **Table 1**. EPEC was grown standing in LB broth overnight at 37°C. *L. reuteri* was cultured standing in an anaerobic cabinet (5% CO_2_, 10% H_2_, and 85% N_2_) overnight at 37°C in MRS broth. Bacteria were spun down and suspended in an equivalent volume of serum-free DMEM medium prior to infection.

**Table 1:** Bacterial strains.

Strain	Host	Reference
***Lactobacillus reuteri***		
ATCC PTA 6475	Human	Oh et al., 2010
ATCC PTA 6475 CmbA^-^	Human	Etzold et al., 2014b
ATCC 55730 (SD2112)	Human	Oh et al., 2010
LMS11-3	Human	Oh et al., 2010
DSM 20016	Human	Oh et al., 2010
ATCC 53608 (1063)	Pig	Oh et al., 2010
1063N	Pig	MacKenzie et al., 2010
100-23C	Rat	Oh et al., 2010
LB54	Chicken	Oh et al., 2010
**EPEC**		
O127:H6 E2348/69	Human	Levine et al., 1978

### Adherence Assays

Cells were seeded out in 24 well plates at a density of 10^5^ cells per well and grown for 6 days (HT-29) or 8 days (LS174T) to full confluence reaching a number of approximately 10^6^ cells per well. For adherence assays, cell monolayers were inoculated with around 5 × 10^7^ *L. reuteri* or EPEC (50 μl of a standing overnight culture of approximately 1 × 10^9^ colony forming units/ml), equaling a multiplicity of infection (MOI) of around 50 bacteria per cell. After incubation for 1 h, cells were washed three times in PBS to remove non-adherent bacteria. Adherent bacteria were quantified by lysing cell monolayers in 1% Triton X-100 for 15 min and plating out serial dilutions of lysates on MRS (*L. reuteri*) or LB agar plates (EPEC). In addition, bacterial inocula and total numbers of EPEC after 1 h of incubation were determined by plating out dilutions of the inocula and combined cell supernatants/lysates, respectively. MRS plates were incubated anaerobically, and LB plates were incubated aerobically at 37°C. Colony forming units (CFU) were determined next day. Adherence was calculated according to the following equation: % adhesion = (number of adherent bacteria/number of inoculated bacteria) × 100.

### Protection Assays

Two different protocols were applied for protection assays. For short-term assays without removal of non-adherent lactobacilli, cell monolayers were pre-incubated with 5 × 10^6^ to 5 × 10^8^ (equal numbers to 100-fold excess, MOI of 5 to 500) *L. reuteri* for 1 or 3 h before 5 × 10^6^ EPEC (MOI of 5) were added for 1 h. To assess protection by adherent lactobacilli, cells were pre-incubated with 5 × 10^8^ *L. reuteri* (MOI of 500) for 4 h, non-adherent bacteria were removed by washing, and cells were further incubated with 5 × 10^6^ EPEC for 1 or 3 h. At the end of the experiment, cell viability and pH of the culture medium were evaluated by Trypan blue stain (0.2%, Sigma) and pH indicator sticks (pH range 4.5–10.0 at 0.5 intervals, Fisher), respectively.

### Polarized *In Vitro* Organ Culture (pIVOC)

This study was performed with approval from the University of East Anglia Faculty of Medicine and Health Ethics Committee (ref 2010/11-030). All samples were provided through the Norwich Biorepository, which has NRES approval (ref 08/h0304/85+5). Biopsy samples from the second part of the duodenum were obtained with informed consent during routine endoscopy of 11 adult patients (48–82 years old). Samples were taken from macroscopically normal areas, transported to the laboratory in IVOC medium and processed within 1 h. Polarized IVOC was performed as described previously (Schüller et al., 2009). Briefly, biopsies were mounted on a membrane filter with the mucosal side facing upward and sandwiched between two Perspex disks with a central aperture. This assembly was then inserted into a Snapwell support (Corning Costar). For adherence assays, biopsies were inoculated with 2 × 10^7^ *L. reuteri* or EPEC on the mucosal side and incubated for 6 h in a 5% CO_2_ atmosphere at 37°C on a rocking platform. For protection assays, biopsies were inoculated with 10^9^ *L. reuteri* for 2 h, non-adherent lactobacilli were removed, and 2 × 10^7^ EPEC were applied for 4 h. At the end of the experiment, biopsies were removed from the Snapwell support, washed three times in PBS to remove non-adherent bacteria and mucus, unless indicated otherwise, and processed for further analysis. For determination of adherent bacteria, biopsies were homogenized with a sterile pestle and lysed as described above.

### Immunofluorescence Staining

Biopsy samples with preserved mucus layer were embedded in OCT compound (Sakura), snap-frozen in a dry ice/ethanol bath and stored at –70°C until use. Serial sections of 7 μm were cut with a Microm HM550 cryostat (Thermo Scientific), picked up on poly L-lysine-coated slides and air-dried. Tissue sections were blocked with 0.5% bovine serum albumin (BSA) in PBS for 20 min. Cells on coverslips were fixed in 3.7% formaldehyde in PBS for 10 min or in methanol/acetone (1:1) for 4 min on ice (for mucus staining) and blocked/permeabilized with 0.1% Tx-100 and 0.5% BSA for 20 min. Cells and cryosections were incubated with primary antibodies (rabbit anti-CmbA (Etzold et al., 2014b), rabbit anti-MUB (MacKenzie et al., 2010), rabbit anti-SRR (kind gift from Donald MacKenzie), goat anti-*E. coli* from abcam, mouse anti-MUC2 from Santa Cruz) for 60 min, washed and incubated with Alexa Fluor-conjugated secondary antibodies (Life Technologies) for 30 min. Cell nuclei and filamentous actin were stained with DAPI (Roche) and FITC-conjugated phalloidin (Sigma), respectively. Samples were mounted in Vectashield (Vector Laboratories) and analyzed using a fluorescence light microscope (Axiovert 200M, Zeiss). Formation of EPEC actin pedestals or microcolonies was quantified from ten random fields of view containing around 70 cells for each experimental condition.

### Scanning Electron Microscopy

Biopsies were either washed in PBS to remove the mucus layer or fixed immediately with preserved mucus layer in 2.5% glutaraldehyde in PBS. Samples were dehydrated through a graded acetone series, dried using tetramethylsilane (Sigma), mounted on aluminum stubs, sputter-coated with gold (Polaron SC7640 sputter coater, Quorum Technologies), and viewed with a JEOL JSM 4900 LV scanning electron microscope.

### Statistical Analysis

All data are shown as means ± standard error of the mean (SEM). Statistical analysis was performed using GraphPad Prism software (version 5). One-way ANOVA with Tukey’s multiple comparisons test was used to determine differences between multiple groups. A *P*-value of <0.05 was considered significant.

## Results

### Adherence of *L. reuteri* to Intestinal Epithelial Cells is Host Strain-Specific

**FIGURE 1:**
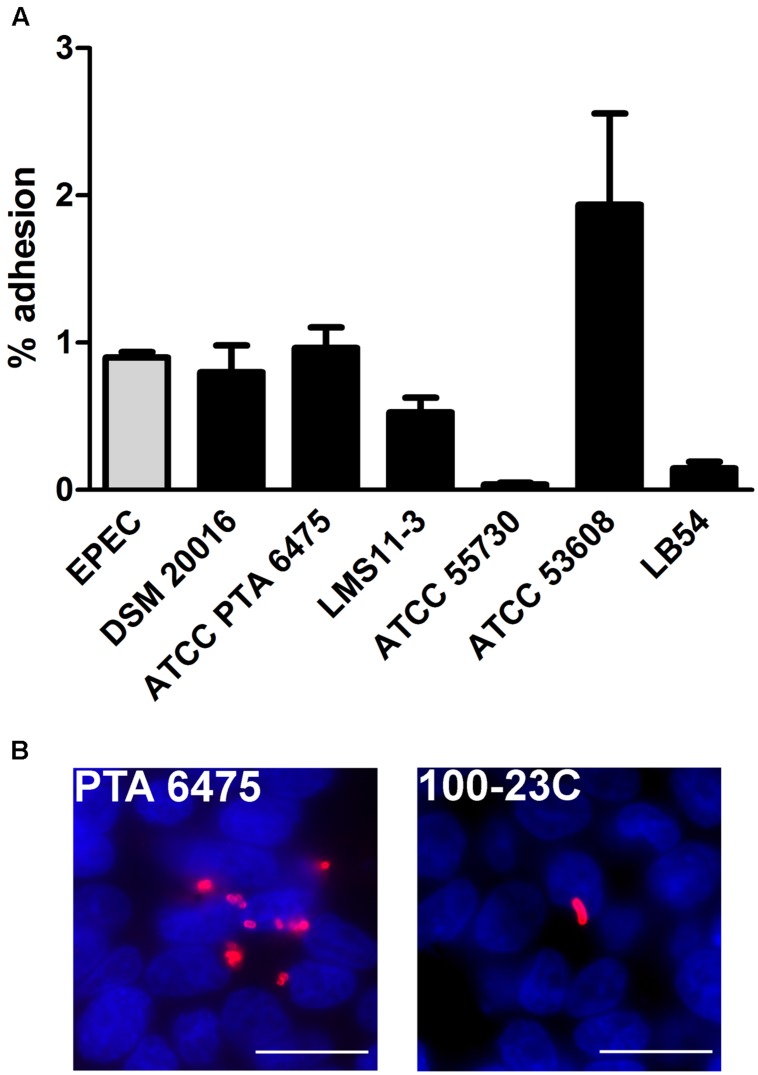
**Adhesion of *L. reuteri* to HT-29 cells is host strain-specific.** HT-29 cells were incubated with *L. reuteri* or EPEC for 1 h, and adherent bacteria were quantified by CFU **(A)** and/or visualized by immunofluorescence staining **(B)**. **(A)** Adhesion is expressed as percentage of adherent bacteria relative to the inoculum. Data are shown as means ± SEM from three independent experiments performed in duplicate. **(B)** *L. reuteri* ATCC PTA 6475 (left) and 100-23C (right) were stained with anti-CmbA and anti-SRR (red), respectively. Cell nuclei were counterstained with DAPI in blue. Shown are representative images from three independent experiments performed in duplicate. Bar = 10 μm.

In order to investigate the adherence properties of *L. reuteri* strains isolated from different hosts and select highest binders, HT-29 cells were first incubated with different *L. reuteri* strains from human, pig, chicken and rat (**Table 1**), or with EPEC prototype strain E2348/69, and adherence was quantified after 1 h. No EPEC replication was detected during this short time period, and 97.0 ± 2.6% of inoculated bacteria (*n* = 5) were recovered after incubation as evaluated by CFU counting. As shown in **Figure 1A**, the *L. reuteri* pig isolate ATCC 53608 showed the highest level of adherence followed by human isolates ATCC PTA 6475 and DSM 20016, which demonstrated comparable binding levels to EPEC. In contrast, the human isolate ATCC 55730 and the LB54 strain from chicken demonstrated low binding, whereas the human strain LMS11-3 exhibited an intermediate adherence potential. Furthermore, the rat isolate 100-23C showed low binding to HT-29 cells compared to ATCC PTA 6475, DSM 20016, LMS11-3, and ATCC 53608 as evaluated by immunofluorescence staining (**Figure 1B**, images shown for ATCC PTA 6475 and 100-23C only) and was therefore not included in the quantitative adherence assay shown in **Figure 1A**. In these assays, it was also noted that ATCC 53608 formed extensive biofilm-like aggregates on cell surfaces (data not shown), as previously reported *in vitro* (MacKenzie et al., 2010). Based on these results, ATCC PTA 6475 (human) and ATCC 53608 (pig) were selected for further studies as they both showed high adherence but different binding phenotypes.

**FIGURE 2:**
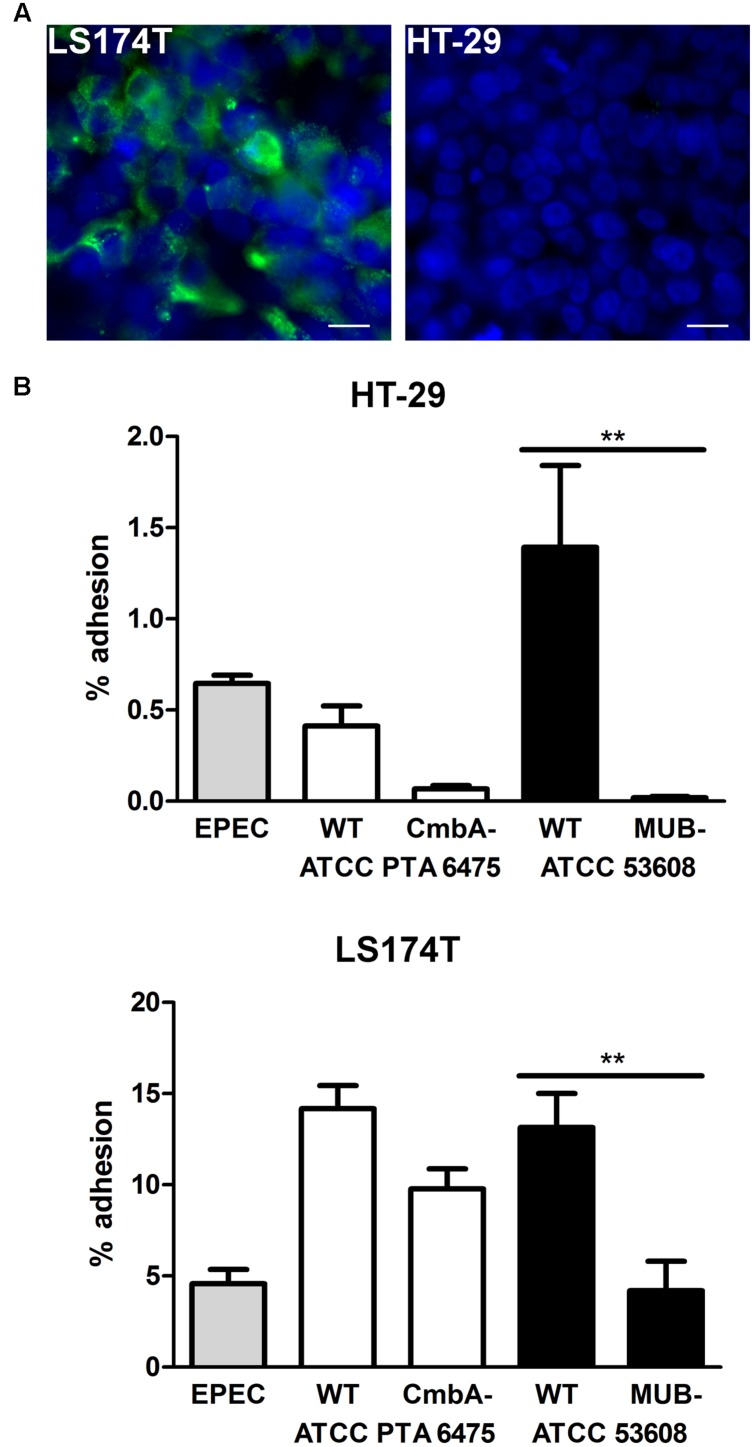
**Mucus binding proteins CmbA and MUB increase *L. reuteri* adhesion to HT-29 and LS174T cells. (A)** Immunofluorescence staining demonstrating MUC2 expression (green) by LS174T but not HT-29 cells. Cell nuclei were labeled in blue. Shown are representative images from three independent experiments performed in duplicate. Bar = 10 μm. **(B)** HT-29 and LS174T cells were incubated with EPEC, or *L. reuteri* strains ATCC PTA 6475 or ATCC 53608 for 1 h. In addition to wild-type strains (WT), isogenic mutants deficient in expression of the adhesin CmbA (ATCC PTA 6475 CmbA-) or MUB (ATCC 53608 MUB-) were included for comparison. Adhesion is expressed as percentage of adherent bacteria relative to the inoculum. Data are shown as means ± SEM from three independent experiments performed in duplicate. ^∗∗^*P* < 0.01.

*Lactobacillus reuteri* binding of these strains to the host is mediated by adhesins on the bacterial surface (Etzold and Juge, 2014) with CmbA (also known as Lar_0958) being present in human isolates (Etzold et al., 2014b; Jensen et al., 2014) whereas MUB is specific for pig isolates (Roos and Jonsson, 2002; MacKenzie et al., 2010; Etzold et al., 2014a). In order to investigate the effect of these adhesins on the binding of the selected strains (ATCC PTA 6475 and ATCC 53608) to intestinal epithelial cells, an ATCC PTA 6475 knockout mutant in CmbA (Etzold et al., 2014b) and an ATCC 53608-derived strain expressing truncated MUB (1063N) (MacKenzie et al., 2010) were compared to the wild-type strains. Here, both HT-29 cells (which do not secrete mucus) and the goblet cell-derived cell line LS174T secreting intestinal mucin MUC2 (**Figure 2A**) were used to assess binding. Quantification of bacterial adherence indicated impaired binding of MUB- and CmbA-deficient strains to both HT-29 and LS174T cells as compared to their respective wild-type strains (**Figure 2B**). In addition, adhesion of all strains tested (including EPEC) was considerably higher on mucus-producing LS174T cells compared to HT-29 cells (**Figure 2B**). Cell viability and pH of the culture medium (pH 7.5) were maintained during all incubations (data not shown).

### *Lactobacillus reuteri* Inhibits EPEC Binding in a Strain- and Cell-Dependent Manner

**FIGURE 3:**
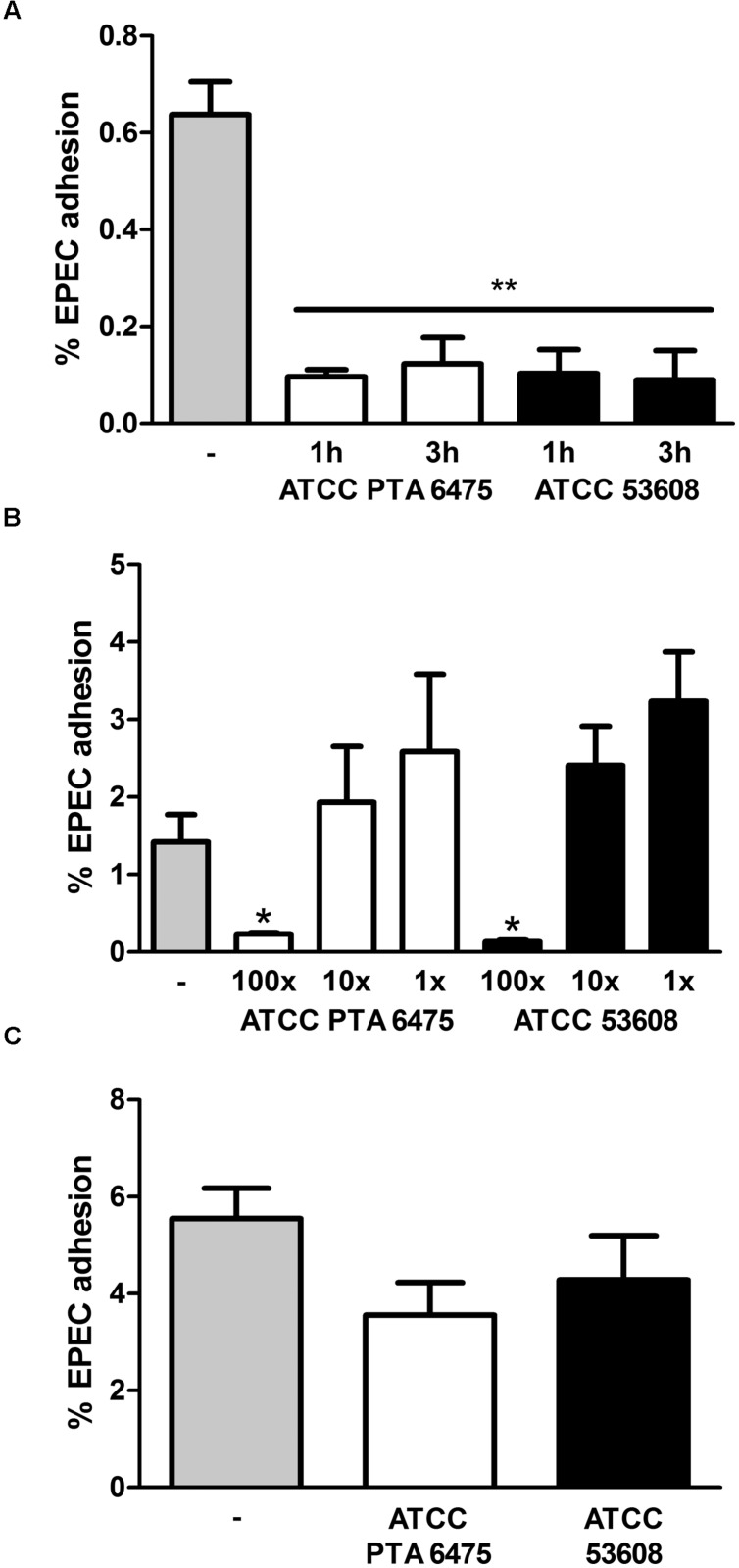
**Pre-incubation with *L. reuteri* ATCC PTA 6475 and ATCC 53608 inhibits EPEC adherence to HT-29 but not LS174T cells. (A)** HT-29 cells were pre-incubated with a 100-fold excess of *L. reuteri* ATCC PTA 6475, ATCC 53608, or left untreated (–) for 1 or 3 h before addition of EPEC for 1 h. **(B)** HT-29 cells were pre-incubated with a 100-fold (100x) or 10-fold (10x) excess or equal numbers (1x) of *L. reuteri* ATCC PTA 6475, ATCC 53608, or left untreated (–) for 1 h before addition of EPEC for 1 h. **(C)** LS174T cells were pre-incubated with a 100-fold excess of ATCC PTA 6475, ATCC 53608 or left untreated (–) for 1 h before addition of EPEC for 1 h. Cell-associated EPEC were quantified by CFU, and adhesion is expressed as percentage of adherent bacteria relative to the inoculum. Data are shown as means ± SEM from three independent experiments performed in duplicate. ^∗^*P* < 0.05, ^∗∗^*P* < 0.01.

To examine whether pre-incubation with *L. reuteri* reduces intestinal EPEC adhesion, HT-29 cells were incubated with a 100-fold excess of *L. reuteri* ATCC PTA 6475 or ATCC 53608 for 1 or 3 h before EPEC was added for another hour. This resulted in significantly reduced EPEC adherence to HT-29 cells (**Figure 3A**). No significant inhibition of EPEC binding was observed using a 10-fold excess or equal numbers of ATCC PTA 6475 or ATCC 53608 (**Figure 3B**). Similar experiments were performed with LS174T cells, and although pre-incubation with a 100-fold excess of *L. reuteri* decreased EPEC adherence to some extent, this was not as pronounced as observed with HT-29 cells and did not reach significance (**Figure 3C**).

**FIGURE 4:**
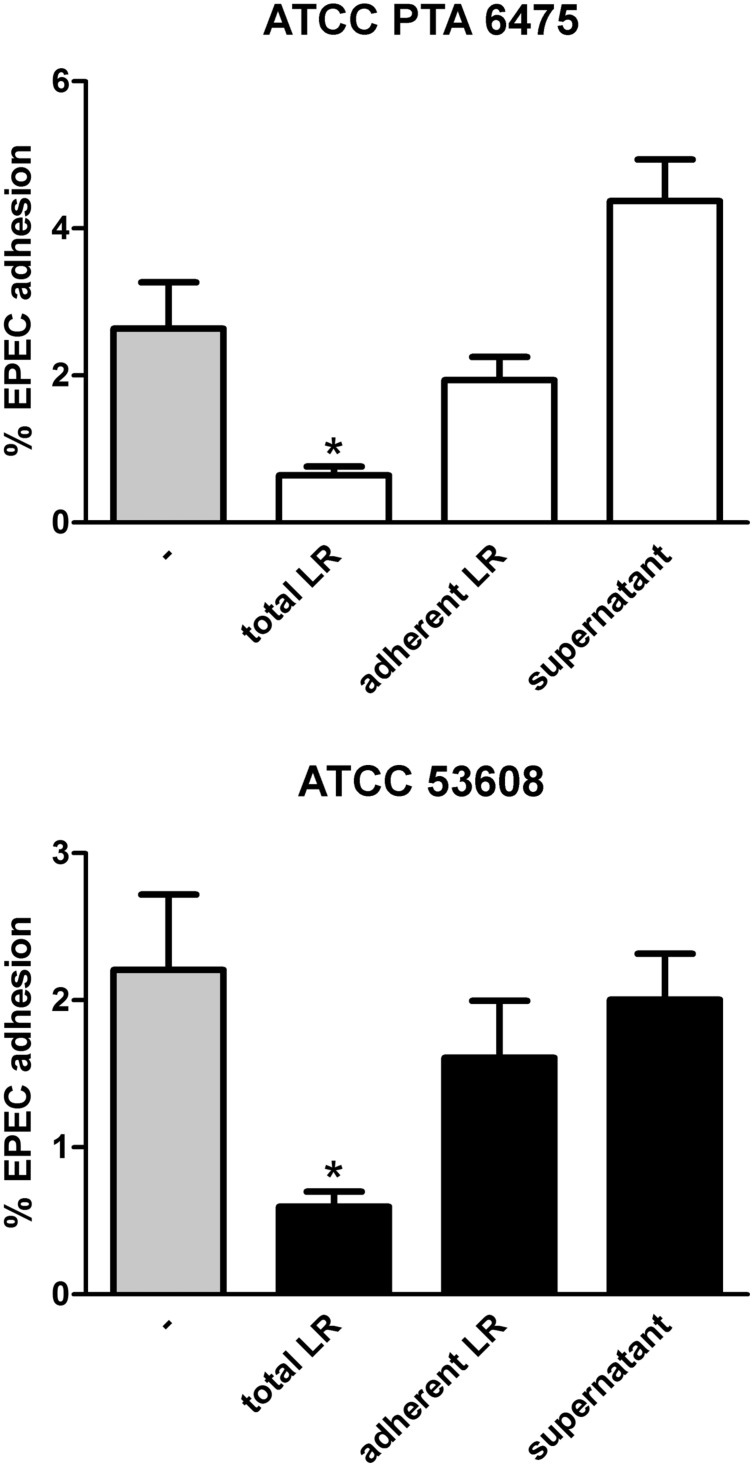
**Reduced EPEC binding to HT-29 cells is independent of *L. reuteri* adherence and secreted molecules.** HT-29 cells were subjected to the following treatments before addition of EPEC for 1 h: pre-incubation with *L. reuteri* for 1 h (total LR), pre-incubation with *L. reuteri* for 2 h and subsequent removal of non-adherent bacteria (adherent LR), pre-incubation with *L. reuteri*-conditioned medium for 2 h (supernatant), or no treatment (–). Cell-associated EPEC were quantified by CFU, and adhesion is expressed as percentage of adherent bacteria relative to the inoculum. Data are shown as means ± SEM from three independent experiments performed in duplicate. ^∗^*P* < 0.05.

Adhesion of bacteria to the mucosa is generally considered as one of the desirable criteria for the selection of probiotics as it promotes intestinal persistence and pathogen exclusion despite peristaltic flow (Servin, 2004). To determine whether reduced EPEC binding was mediated by *L. reuteri* adhering to the epithelium, HT-29 cells were first incubated with a 100-fold excess of *L. reuteri* ATCC PTA 6475 or ATCC 53608 for 2 h, and in contrast to experiments described above, non-adherent bacteria were removed by washing before addition of EPEC for 1 h. In addition, to test the potential impact of released antimicrobial components in the supernatant, HT-29 cells were incubated with *L. reuteri*-conditioned medium (cultured with HT-29 cells for 2 h) and EPEC for 1 h. As shown in **Figure 4**, none of these treatments resulted in significant reduction of EPEC adherence.

**FIGURE 5:**
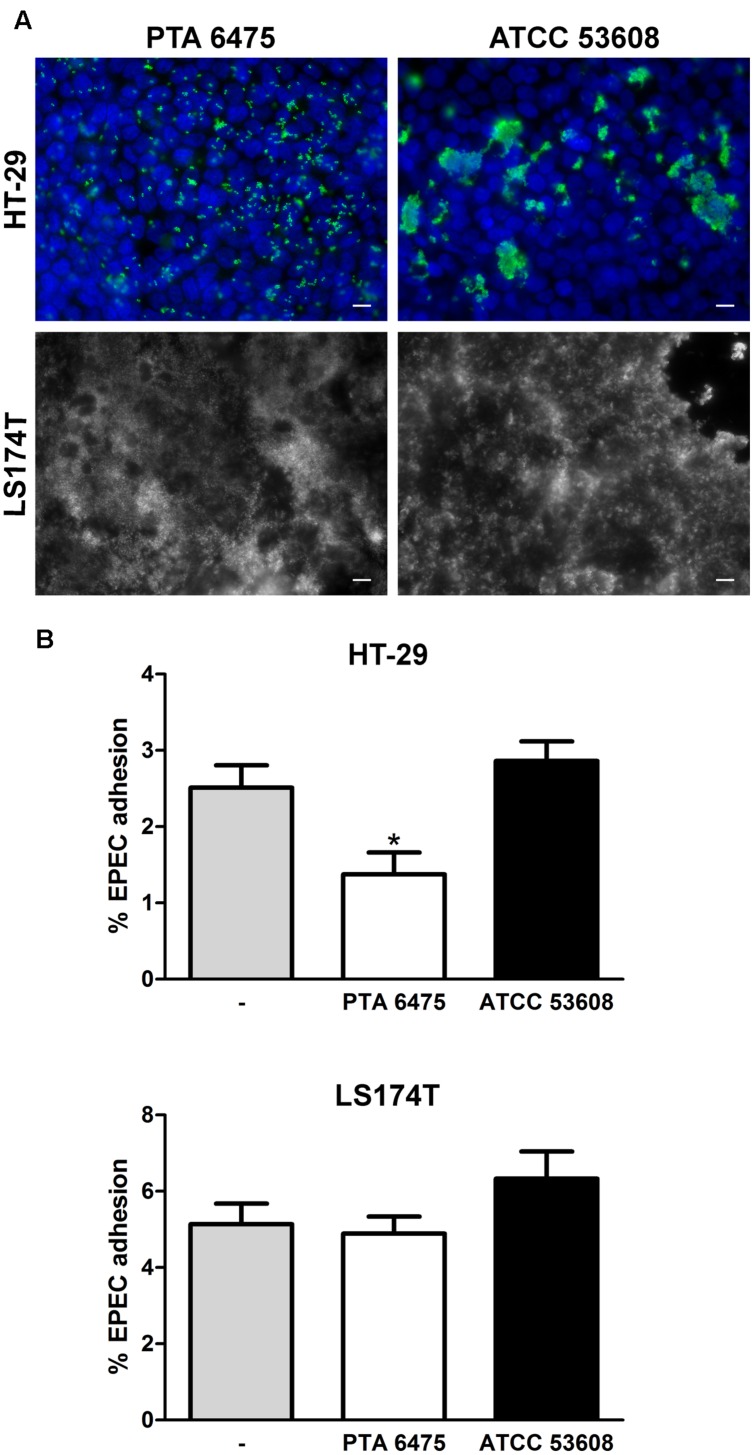
**Adherent *L. reuteri* ATCC PTA 6475 inhibits EPEC binding to HT-29 but not LS174T cells. (A)** HT-29 and LS174T cells were incubated with *L. reuteri* ATCC PTA 6475 or ATCC 53608 for 4 h. Adherent *L. reuteri* were visualized by immunofluorescence staining with anti-CmbA (PTA 6475) or anti-MUB (ATCC 53608) in green. Cell nuclei were counterstained with DAPI (blue). Merged color images of both channels are shown for HT-29 cells, whereas black-and-white images of the green channel (*L. reuteri* staining) are displayed for confluent LS174T cells for greater clarity. Shown are representative images from three independent experiments performed in duplicate. Bar = 10 μm. **(B)** HT-29 and LS174T cells were pre-incubated with ATCC PTA 6475, ATCC 53608, or left untreated (–) for 4 h, non-adherent bacteria were removed, and EPEC was added for 1 h. Cell-associated EPEC were quantified by CFU, and adhesion is expressed as percentage of adherent bacteria relative to the inoculum. Data are shown as means ± SEM from three independent experiments performed in duplicate. ^∗^*P* < 0.05.

**FIGURE 6:**
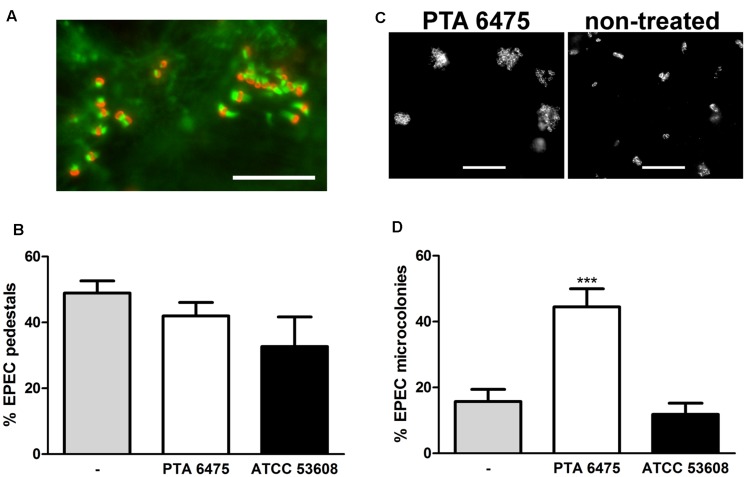
**Adherent *L. reuteri* ATCC PTA 6475 enhances EPEC microcolony formation on LS174T cells.** LS174T cells were pre-incubated with *L. reuteri* ATCC PTA 6475, ATCC 53608, or left untreated (–) for 4 h, non-adherent bacteria were removed, and EPEC was added for 3 h. **(A)** EPEC forms actin pedestals on LS174T cells as visualized by immunofluorescence staining (EPEC stained in red and polymerized actin in green). Representative image from four independent experiments performed in duplicate. Bar = 10 μm. **(B)** Quantification of EPEC bacteria associated with actin pedestals by counting. EPEC pedestal formation is expressed as percentage of bacteria associated with polymerized actin relative to the total number of adherent EPEC observed. Data are shown as means ± SEM from four independent experiments performed in duplicate. **(C)** Pre-incubation with *L. reuteri* ATCC PTA 6475 results in enhanced EPEC microcolony formation compared with non-treated samples. Adherent EPEC were visualized by immunofluorescence staining. Representative images from four independent experiments performed in duplicate. Bar = 10 μm. **(D)** Quantification of EPEC microcolony formation (five or more bacteria) by counting. Microcolony formation is expressed as percentage of bacteria associated with microcolonies relative to the total number of adherent EPEC observed. Data are shown as means ± SEM from four independent experiments performed in duplicate. ^∗∗∗^*P* <0.001.

Increasing the pre-incubation period to 4 h promoted *L. reuteri* adherence as shown by immunofluorescence staining of ATCC PTA 6475 and ATCC 53608 bound to HT-29 and LS174T cells (**Figure 5A**). Non-adherent bacteria were removed by washing, and cells were subsequently incubated with EPEC for 1 h. Adherent ATCC PTA 6475 but not ATCC 53608 significantly reduced EPEC binding to HT-29 cells (**Figure 5B**). In contrast, no significant inhibition of EPEC adhesion was observed using LS174T cells (**Figures 5A,B**) despite the higher *L. reuteri* binding to this cell type as reported above. As LS174T cells secrete mucus, it is likely that a large proportion of EPEC is located within the mucus layer rather than bound to the epithelial cell membrane. Therefore, CFU from cell lysates might not accurately reflect the number of EPEC bacteria adhering to the cell surface. To this aim, the protection assay was modified, and the incubation time with EPEC was extended to 3 h to allow the formation of A/E lesions. Cell-bound EPEC were subsequently identified by fluorescent actin staining (**Figure 6A**), and the number of EPEC associated with actin pedestals was determined. Quantification of actin-linked versus total cell-associated bacteria revealed that there was no significant difference in A/E lesion formation on cells pre-incubated with *L. reuteri* and non-treated controls (**Figure 6B**). However, larger clusters of A/E bacteria were observed on LS174T cells pre-incubated with ATCC PTA 6475 (**Figure 6C**), and quantification of microcolony formation (five or more bacteria per colony) at a higher magnification confirmed that pre-incubation with ATCC PTA 6475 significantly increased the number of A/E microcolonies as compared to ATCC 53608 and non-treated controls (**Figure 6D**). Cell viability and pH of the culture medium (pH 7.5) were maintained during all incubations (data not shown).

### *Lactobacillus reuteri* Binds to the Mucus Layer and Decreases EPEC Adherence to Human Small Intestinal Biopsies

**FIGURE 7:**
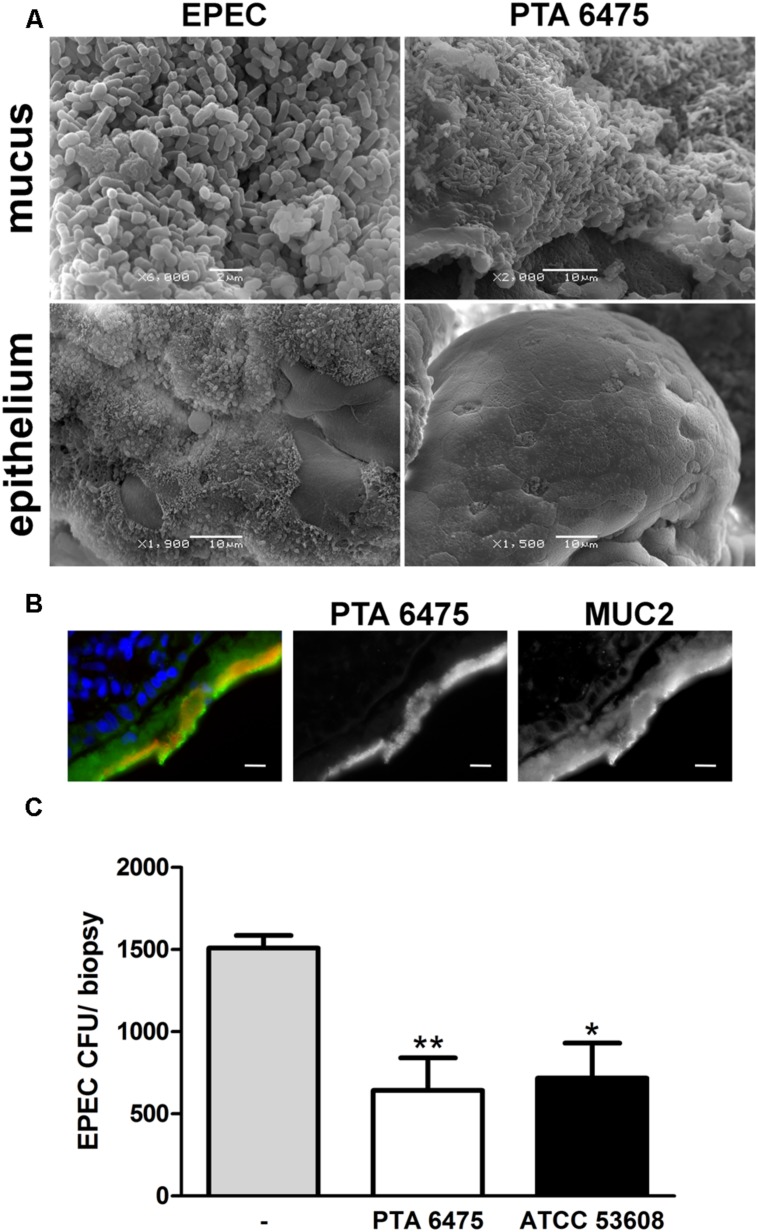
***L. reuteri* is localized in the mucus layer and inhibits EPEC binding to human duodenal biopsy epithelium. (A)** Scanning electron microscopy of duodenal biopsies incubated with EPEC or *L. reuteri* ATCC PTA 6475 for 6 h. Biopsies were either fixed with mucus layer or washed beforehand to expose the epithelium. Shown are representative images from three independent experiments performed in duplicate. **(B)** Immunofluorescence staining of duodenal biopsies incubated with *L. reuteri* ATCC PTA 6475 for 6 h. Cryosections were stained for MUC2 (green), lactobacilli (anti-CmbA, red) and cell nuclei (blue). Shown are representative images from four independent experiments performed in duplicate. Bar = 10 μm. **(C)** Duodenal biopsies were pre-incubated with *L. reuteri* ATCC PTA 6475, ATCC 53608, or left untreated (–) for 2 h, non-adherent bacteria were removed, and EPEC was added for 4 h. Cell-bound EPEC were quantified by CFU, and adherence is expressed as CFU per biopsy. Data are shown as means ± SEM from four independent experiments performed in duplicate. ^∗^*P* < 0.05, ^∗∗^*P* < 0.01.

To examine whether the results obtained on cancer-derived cell lines could be translated to human intestinal tissue, a polarized *ex vivo* model using human small intestinal biopsies was employed. Polarized *in vitro* organ culture (pIVOC) restricts bacterial access to a defined mucosal surface area and therefore allows quantification of bacterial adhesion (Schüller et al., 2009). Adult endoscopic biopsies from the duodenum were incubated with *L. reuteri* ATCC PTA 6475, ATCC 53608, or EPEC and incubated for 6 h. Biopsies were then either fixed with mucus layer or washed first to remove mucus and expose the epithelium, and bacterial adherence was visualized by scanning electron microscopy. As shown in **Figure 7A**, *L. reuteri* was observed in the mucus layer but not on the epithelium (images shown for ATCC PTA 6475 only), whereas EPEC was present in both the mucus layer and on the epithelium where A/E lesions were formed. None of the treatments visibly affected epithelial integrity, and no cell extrusion was observed (**Figure 7A**). The confinement of *L. reuteri* to the mucus layer was also confirmed by immunofluorescence staining of biopsy cryosections (**Figure 7B**, images shown for ATCC PTA 6475 only). For protection assays, biopsies were pre-incubated with a 50-fold excess of *L. reuteri* for 2 h, non-adherent bacteria were removed, and EPEC was added for another 4 h to enable penetration of the mucus layer and epithelial binding. At the end of the experiment, the mucus layer was removed by washing, and EPEC adherence to the epithelium was quantified. As demonstrated in **Figure 7C**, pre-incubation with ATCC PTA 6475 or ATCC 53608 significantly reduced EPEC adherence to duodenal biopsy epithelium.

## Discussion

The gut symbiont *L. reuteri* is unique among probiotic bacteria in that it resides in a range of hosts (including mammals and birds) and can protect against disease in the species of origin (Casas and Dobrogosz, 2000). Previous studies have demonstrated host adaptation of *L. reuteri* with evolution of host-specific binding proteins which enable rodent but not human, pig, or chicken isolates to colonize the gut of mice (Frese et al., 2011). Similarly, our results showed good adherence of human and pig isolates versus reduced binding of rat and chicken isolates to human HT-29 cells, with the exception of the human isolate ATCC 55730. This was surprising as ATCC 55730 and its plasmid-free derivative DSM17938 have been widely used in probiotic trials (Szajewska et al., 2014), and colonization of the human stomach and small intestine has been shown (Valeur et al., 2004).

*Lactobacillus reuteri* expresses several adhesins such as the mucus-binding proteins MUB (Roos and Jonsson, 2002), CmbA (Etzold et al., 2014b; Jensen et al., 2014), and MapA (Miyoshi et al., 2006) with the latter two also mediating adherence to Caco-2 cells. Here, we show that MUB and CmbA increase *L. reuteri* adherence to HT-29 cells which, like Caco-2 cells, do not produce MUC2, the main secreted mucin in the human intestine (van Klinken et al., 1996; Johansson et al., 2011). A similar effect, although less pronounced, was also observed in MUC2-secreting LS174T cells indicating binding of MUB and CmbA to both human epithelial cell surface molecules and secreted mucins. Interestingly, *L. reuteri* strains and EPEC adhered considerably better to LS174T compared with HT-29 cells. This might reflect a higher bacterial binding affinity to mucus compared with the epithelial surface, as previously demonstrated for commonly used probiotic strains on human intestinal tissue pieces and mucus (Ouwehand et al., 2002).

One suggested mechanism of probiotic protection is competitive exclusion where probiotics and pathogens compete for binding sites on the epithelial surface. This generally requires pre-incubation (preventive treatment) as pathogen displacement by lactobacilli is seldom observed (Lebeer et al., 2008). While no such studies have been performed on *L. reuteri* and EPEC so far, *L. acidophilus* has been shown to inhibit EPEC binding to Caco-2 cells when pre-incubated at equal numbers (Bernet et al., 1994). Similarly, pre-incubation with *L. rhamnosus* or *L. acidophilus* at a 1,000-fold or 10-fold excess, respectively, reduced EPEC adherence to polarized T84 cells (Sherman et al., 2005), and *L. rhamnosus* inhibited EPEC adherence to Caco-2 cells when used at a 10-fold excess (Forestier et al., 2001). In addition, pre-incubation with *L. acidophilus* or *L. rhamnosus* inhibited adherence of the related A/E pathogen enterohemorrhagic *E. coli* to HT-29 cells at a 1,000-fold excess (Kim et al., 2008). Therefore, our findings that a pre-incubation with *L. reuteri* at a 100-fold excess decreases EPEC adherence to HT-29 cells, are in agreement with previous studies on other *Lactobacillus* species. In the conditions of the short term protection assay, the effect was not solely reliant on the adhesion of *L. reuteri* strains to the cell surface, suggesting that steric hindrance may also play a role (Lebeer et al., 2008).

Other probiotic mechanisms of microbe–microbe interactions apart from competitive exclusion include production of antimicrobial compounds. Previous studies have demonstrated that lactic acid produced by lactobacilli has a bactericidal effect against EHEC and *Salmonella* Typhimurium (Ogawa et al., 2001; De Keersmaecker et al., 2006). In addition to lactic acid, some *L. reuteri* strains produce the antimicrobial substance reuterin during glycerol fermentation (Talarico et al., 1988). These compounds did not appear to be involved in the observed inhibitory effect in our experimental model as *L. reuteri*-conditioned media were not sufficient to reduce EPEC adherence.

In contrast to static cell culture models, peristaltic movement and flow rapidly clear non-adherent bacteria from the gut (Servin, 2004). Modification of the cell culture model to allow efficient adherence of *L. reuteri* supported two mechanisms to reduce EPEC infection which were evident for strain ATCC PTA 6475 only. Firstly, adherent ATCC PTA 6475 reduced EPEC adhesion to HT-29 cells suggesting blockage of specific binding sites. This effect was not observed for ATCC 53608 which showed aggregative binding to distinct cells, whereas ATCC PTA 6475 was uniformly distributed. Auto-aggregation of ATCC 53608 might prevent efficient spread and blockage of cell surface receptors in contrast to non-aggregating ATCC PTA 6475. In contrast to HT-29 cells, no inhibition of EPEC binding was observed on LS174T cells. This difference could be explained by direct contact of *L. reuteri* with epithelial EPEC receptors on HT-29 cells while contact with the mucus layer on LS174T cells would not directly compete with EPEC adherence to the epithelium. However, a significantly higher number of EPEC microcolonies was observed with ATCC PTA 6475 compared with LS174T cells pre-incubated with ATCC 53608 and the non-treated control. Although both *L. reuteri* strains appeared to bind similarly to LS174T cells, different adherence to the epithelial surface (as opposed to the overlying mucus), as observed on HT-29 cells, may provide a second mechanism by which ATCC PTA 6475 could potentially inhibit EPEC microcolony dispersal and cell-to-cell spread by blockage of cell surface receptors.

Furthermore, both ATCC PTA 6475 and ATCC 53608 significantly inhibited EPEC binding to duodenal biopsy epithelium. As both *L. reuteri* strains were shown to be confined to the mucus layer, this suggests that, in presence of a preserved mucus layer, the inhibitory effect of *L. reuteri* strains is exerted at the mucus interface rather than the epithelial surface. The exact mechanism of this effect still remains to be elucidated, but it is conceivable that *L. reuteri* binding to the mucus layer could result in a stronger physical barrier against EPEC infection. Contrasting results in LS174T cells and duodenal biopsy tissue might be explained by differences in mucus organization and composition. Notably, LS174T cells present a “goblet cell-like” phenotype and produce MUC2 but also demonstrate an aberrant secretion of human gallbladder mucin and gastric MUC5AC often associated with colon cancer (van Klinken et al., 1996; Bartman et al., 1999). This is in contrast to human duodenal biopsy mucosa which presents a single layer of secreted MUC2.

In summary, our studies have demonstrated that *L. reuteri* can reduce EPEC infection by several mechanisms which include competitive exclusion at the mucus or epithelial level or *via* potential inhibition of microcolony dispersal and cell-to-cell spread. These effects were strain-specific and dependent on the intestinal model system used, highlighting the need for a careful choice of experimental models when selecting potential probiotic strains. Alterations in the mucus layer have been reported in a number of infectious and inflammatory diseases, often facilitating bacterial access to the epithelial surface. Whether individuals present with a healthy or compromised mucus layer may therefore influence the rationale for selecting specific probiotic strains.

## Author Contributions

AW, DM, NJ, and SS designed the study. DM and NJ provided bacterial strains, cell lines, and antibodies. VC and SW provided human tissue samples. AW and CH performed the experimental work and analyzed the data. SS and AW prepared the manuscript, and NJ and DM contributed to the final manuscript.

## Conflict of Interest Statement

The authors declare that the research was conducted in the absence of any commercial or financial relationships that could be construed as a potential conflict of interest.
